# Corrigendum

**DOI:** 10.1002/ehf2.13116

**Published:** 2020-12-03

In the paper by Yamamoto *et al*.,^1^ Yusuke Yoshikawa should have been included as a co‐author. The correct list of authors is as follows:

Erika Yamamoto^1^, Takao Kato^1*^, Hidenori Yaku^1^, Takeshi Morimoto^2^, Yasutaka Inuzuka^3^, Yodo Tamaki^4^, Neiko Ozasa^1^, Yusuke Yoshikawa^1^, Takeshi Kitai^5^, Ryoji Taniguchi^6^, Moritake Iguchi^7^, Masashi Kato^8^, Mamoru Takahashi^9^, Toshikazu Jinnai^10^, Tomoyuki Ikeda^11^, Yoshihiro Himura^11^, Kazuya Nagao^12^, Takafumi Kawai^13^, Akihiro Komasa^14^, Ryusuke Nishikawa^15^, Yuichi Kawase^16^, Takashi Morinaga^17^, Mitsunori Kawato^18^, Yuta Seko^19^, Mamoru Toyofuku^20^, Yutaka Furukawa^6^, Yoshihisa Nakagawa^5^, Kenji Ando^17^, Kazushige Kadota^16^, Satoshi Shizuta^1^, Koh Ono^1^, Yukihito Sato^7^, Koichiro Kuwahara^21^, Takeshi Kimura on behalf of the KCHF Study Investigators.
